# Validation of retroactively derived T1 relaxation values from 3D T1-weighted images with clinical and MRI measures of disability in multiple sclerosis

**DOI:** 10.1371/journal.pone.0323898

**Published:** 2025-05-19

**Authors:** Griffin Young, Vivian S. Nguyen, Quentin Howlett-Prieto, Amanda Frisosky Abuaf, Timothy J. Carroll, Adil Javed

**Affiliations:** 1 Department of Radiology, The University of Chicago, Chicago, Illinois, United States of America; 2 Department of Neurology, The University of Chicago, Chicago, Illinois, United States of America; 3 Department of Neurology, The University of Wisconsin, Madison, Wisconsin, United States of America; King's College Hospital NHS Trust: King's College Hospital NHS Foundation Trust, UNITED KINGDOM OF GREAT BRITAIN AND NORTHERN IRELAND

## Abstract

**Background:**

Quantitative T1 mapping is a valuable technique for assessing tissue injury in multiple sclerosis (MS) lesions. We previously introduced a novel methodology for converting high-resolution anatomical 3D T1-weighted (T1W) images into parametric T1 relaxometry maps. Herein, we correlate MS lesion pathology as quantified by retroactive T1 mapping with clinical and MRI metrics of disability and with magnetization transfer ratio (MTR).

**Methods:**

38 subjects with relapsing-remitting MS (RRMS) were examined, contributing to 587 unique lesions for analysis. T1 and MTR values were compared using correlation statistics. Univariate correlations between lesional T1 or MTR and Expanded Disability Status Scale (EDSS) were examined using Spearman’s rho (ρ), and for disease duration and brain parenchymal fraction (BPF), Pearson’s r. Mean T1 values of lesions were compared across different categories of EDSS severity using Kruskal-Wallis test. Ordinal regression model was used to assess the association between EDSS and T1 values of select brain regions.

**Results:**

The mean T1 of lesions showed a high correlation with - MTR, r = 0.68. T2 lesion volumes stratified based on different T1 thresholds showed a significant correlation with MS disease metrics: lesion volume threshold at 700 < T1 < 900 was correlated with disease duration (r = 0.34, p = 0.04) and BPF (r = -0.47, p = 0.003); lesion volume threshold at 900 < T1 < 1100 was correlated with EDSS (ρ = 0.42, p = 0.01), disease duration (r = 0.45, p = 0.01), and BPF (r = -0.56, p < 0.001); lesion volume threshold at T1 > 1100 was correlated with EDSS (ρ = 0.41, p = 0.01), disease duration (r = 0.45, p = 0.001), and BPF (r = -0.51, p < 0.001). T1 values at the 25^th^, 50^th^, and 75^th^ percentiles significantly correlated with BPF (r = -0.41, p = 0.01; r = -0.41, p = 0.01; r = -0.38, p = 0.02). MTR showed a significant correlation with EDSS but not with disease duration or BPF. Mean T1 values in the NAWM showed a significant association with EDSS (coefficient = 0.03, pseudo R^2^ = 0.07, p = 0.03).

**Conclusions:**

We provide clinical validation of retroactive T1 mapping as a complementary post-processing technique for monitoring disease activity and disability progression in MS.

## Introduction

Multiple sclerosis (MS) is an autoimmune inflammatory disease characterized by multifocal demyelinating lesions in the brain and spinal cord. There is also microstructural damage in the non-lesional normal appearing white and gray matter (NAWM, NAGM). MRI is an indispensable tool for examining MS lesions, however, conventional MR imaging used in clinical practice has several drawbacks, largely in terms of its sensitivity for analyzing tissue injury within MS lesions. Microstructural damage in the NAWM and NAGM also remains clandestine on conventional MR imaging. Histological analysis of MS lesion pathology evinces a significant heterogeneity in the various types of MS lesions, from acute to chronic and from active to inactive, which is only partially captured on conventional MR imaging [1]. Advanced MRI techniques, such as T1 relaxation mapping (T1), magnetization transfer imaging (MTI), diffusion tensor imaging (DTI), and magnetic resonance spectroscopy (MRS) among others, however, offer more sensitive and quantitative analysis of different types of MS lesions, which culminate into improved correlation of MS lesions with clinical measures of disability [2–10].

Given the advantages of incorporating advanced MRI metrics in examining MS lesions, we have previously described a method for generating T1 maps retroactively from high-resolution T1-weighted (T1W) images such as MPRAGE [11]. Despite being a derivative, the resulting T1 maps maintain an isotropic resolution, a benefit not typically seen in parametric T1 imaging methods like inversion recovery. The MS lesion contrast also appears more robust on the resultant T1 maps than the original T1W image. The mean T1 values of MS lesions from these retroactive T1 maps show a high correlation of r = 0.81and bias of 10.17% in comparison with the T1 maps derived from standard T1 relaxometry methodology, i.e., IR-FLASH [12]. Therefore, the main advantage of retroactive T1 mapping is that it allows for capturing quantitative data from high-resolution T1W maps using a post-processing methodology that maintains high fidelity to the natively acquired T1 maps. Furthermore, native T1 mapping techniques or other non-conventional imaging methods for that matter are difficult to implement broadly because they require additional scanning time, local familiarity with more complicated protocols, complexities in synchronization of scanning protocols across different MRI platforms, and greater dependence on MRI software and hardware upgrades. Our retroactive methodology bypasses these concerns since it is derived from T1W images which are much easier to implement and harmonize across different scanners.

The goal of the present study was to further validate the usefulness of retroactive T1 mapping against clinical and MRI measures of MS disability. We also compare the performance of retroactive T1 values against MTR for gauging clinical measures of MS related disability to reinforce our approach since MTR is an analogous MRI technique for classifying MS lesions. The specific hypotheses tested are: 1) T1 and MTR values of MS lesions have a high correlation; 2) T1 and MTR values of lesions correlate with MS disease metrics such as EDSS, disease duration, and BPF; 3) T1 and MTR values of NAWM, cortical gray matter, and thalamus correlate with EDSS. The main goal of this study was to demonstrate the usefulness of retroactive T1 mapping as a suitable biomarker for assessing microstructural changes in MS lesions and normal-appearing tissue using high-resolution T1W images that are already available, such as those from clinical practice or research trials.

## Methods

### Subjects

In this cross-sectional study, 38 subjects with relapsing-remitting MS (RRMS) were examined. MS patients were included if they were clinically stable and had no new lesions on MRI for at least 1 year prior to enrollment. All subjects were on a high-efficacy treatment for at least 1 year prior to inclusion. Subjects with active disease on MRI, recent use of steroids, or initiation of a disease modifying treatment (DMT) < 1 year were excluded. All data were anonymized prior to analysis. The study was approved by the Institutional Review Board (IRB) at University of Chicago Medical Center under the protocol numbers 15–1042 and 22–1723. This study conformed to the ethical standards of the 1964 Declaration of Helsinki. All subjects signed an IRB approved written consent form to participate in the research study. MRI data was captured during the enrollment period from 07/01/2022 to 08/01/22 and 12/08/22 to 03/03/2024, within the IRB approval dates. Data were collected only once per subject during this period. The baseline demographic, clinical, and imaging characteristics of all subjects are shown in Table 1.

**Table 1:** Demographic, clinical, and MRI characteristics of the MS subjects.

Metric	Value
Age (median, IQR), years	43 (37- 49.5)
Male/Female	7/31
Baseline EDSS (median, IQR)	2.5 (2.0 - 3.5)
Disease Duration, years (median, IQR)	11 (5.5 - 16.5)
Total T2 Lesion Load cm^3^ (median, IQR)	3.50 (2.25–7.45)
BPF (median, IQR)	0.79 (0.77–0.82)

Abbreviations: IQR, interquartile range; EDSS, expanded disability status score; BPF, brain parenchymal fraction.

### MR imaging data acquisition

All images were acquired on a Philips 3T 32-channel head coil (Philips Medical Systems, Best, The Netherlands). The protocol included: 3D Turbo Field Echo (TFE) (TR = 8 ms, TE = 3.5 ms, TI = 960 ms, Flip Angle = 8°, Echo Train Length = 228, voxel size = 1x1x1 mm^3^ size = 240x240x155); Proton Density Weighted FFE with and without MT saturation pulse (RF field strength 9.3 μT, TR = 48.11 ms, TE = 5 ms, Flip Angle = 5°, Echo Train Length = 1, voxel size = 1x1x3 mm3, matrix size = 256x256x120); FLAIR (TR = 7500 ms, TE = 135 ms, TI = 2500 ms, Echo Train Length = 34, 120° refocusing pulse, voxel dimensions = 0.5x0.5x3, matrix size, 480x480x59).

### Image processing

All images were corrected for field inhomogeneity and skull stripped in MATLAB (v2022a, MathWorks, Natick, MA, USA) with SPMv12 (Wellcome Centre for Human Neuroimaging, University College London, UK). T1 maps were generated from the 3D TFE images as detailed previously [12]. MTR images were derived pixel-by-pixel according to the equation: MTR= ($M_{0}-M_{s})/\;M_{0}x100,$ where $M_{0}$is the mean signal intensity for a given pixel without the saturation pulse and $M_{s}$ is the mean signal intensity for the same pixel with the saturation pulse.

Cortical and subcortical gray matter structures were segmented from the high-resolution anatomical images using FreeSurfer image processing pipeline v7.4 (https://surfer.nmr.mgh.harvard.edu/fswiki/Samseg). T1 maps, MTR, tissue segmentations, and anatomical 3DTFE images were registered to their respective FLAIR images using the Advanced Normalization Tools (ANTs) SyNQuick registration module (https://github.com/ANTsX/ANTs/wiki). Only whole integer labels were assessed to account for partial volume error in the segmentation registration. All voxels that displayed decimal values due to voxel averaging were discarded to remove any potential label uncertainties at the mask boundaries. For MS lesion analysis, T2 FLAIR hyperintensities were manually segmented by trained experts via 3D Slicer (https://www.slicer.org) [13]. Regions of interest (ROIs) for analysis included MS lesions, NAWM, cortical gray matter, and thalamus. The brain parenchymal fraction (BPF) for each patient was calculated as: BPF= (WM + GM)/(WM + GM + CSF), where white matter (WM), gray matter (GM), and cerebrospinal fluid (CSF) are segmented brain matter volumes after lesion filling.

### Statistical analyses

Statistical analyses were performed using either MATLAB (v2022a, MathWorks, Natick, MA, USA) or R package. To avoid measurement noise due to partial volume signal averaging, cerebrospinal fluid (CSF) masks were dilated by one voxel and removed from the T1 and MTR masks before analysis.

For comparison of T1 and MTR values of MS lesions, correlation plots, Pearson’s r coefficient, and Bland-Altman analysis were performed. Univariate correlations between lesional T1 or MTR measures and EDSS were examined using Spearman’s rho and for disease duration and BPF, Pearson’s r. T1 values of lesions were assessed in two different ways: 1) T1 values of lesions were expressed in terms of 25^th^, 50^th^, 75^th^ percentiles, normalized histogram peak height, and mode, as is de rigueur in MTR studies [14–15]; 2) T2 lesion volumes were also stratified based on T1 intensity thresholds and correlated with EDSS, disease duration, and BPF. Mean T1 of lesions was also compared across different categories of EDSS severity using Kruskal-Wallis test, with low severity of EDSS between 0–2, moderate 2.5–4, and high 4.5–6.5. A p-value < 0.02 was considered to be significant after applying Bonferroni correction. EDSS scores were categorized into disease severity subgroups based on the definitions of functional system (FS) scores in accordance with the Kurtzke Expanded Disability Status Scale [16], with subjects with a score of < 2.0 having minimal disability in at least one FS score, 2.5–4.0 having one or more FS score in the moderate range, and above 4.5 having one or more FS scores in the severe range. EDSS scores > 6.5 were excluded because the scale plateaus above this range and hence correlation and predictive statistics lose sensitivity, as is done in most clinical trials of MS. Our approach of categorizing EDSS scores is pragmatic and based on EDSS FS definitions. Other studies have taken similar albeit slightly different approach, however there is no well-defined operational definition for categorizing EDSS severity scores [17–18].

To assess whether T1 or MTR metrics in the normal-appearing white and select gray matter structures influence EDSS, ordinal regressions were used with age as a nuisance covariate. This was done to reduce any effects of age on T1 values, although previous studies have shown minimal if any variations in T1 values within the age range of 37–50, which was used in our study [19–20]. NAWM and NAGM structures such as the thalamus and cortical gray matter (CGM) were selected *a priori* for analysis given their prominence in MS, as pathology in these regions indicates disease progression [21]. Given the limited number of comparisons, the importance of the selected structures in MS disease process, and the exploratory nature of the analysis in the normal appearing tissue, p values are reported without Bonferroni correction.

## Results

### Demographics

The median age of MS patients was 43 years (IQR 37–49.5), median disease duration 11years (IQR 5.5–16.5), median baseline EDSS score 2.5 (IQR 2.0–3.5), median T2 lesion volume 3.50 cm^3^ (IQR 2.25–7.45), and median BPF 0.78 (IQR 0.77–0.82). The demographic, clinical, and MRI characteristics of all patients are summarized in Table 1.

### T1 and MTR agreement

**Fig 1:**
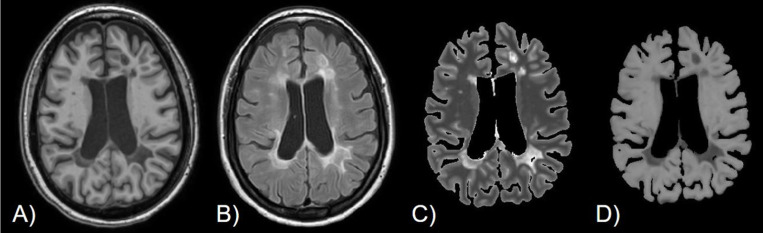
Representative depiction of: A) T1-weighted B) T2 FLAIR, C) retroactive T1 map, and D) MTR map.

Representative 3D T1W (A), T2 FLAIR (B), retroactive T1 (C), and MTR (D) images are depicted in Fig 1. The T2 hyperintensities seen on FLAIR images are well delineated in the corresponding quantitative T1 and MTR maps. The T1 map (C) shows MS lesions with high contrast and definition.

**Fig 2:**
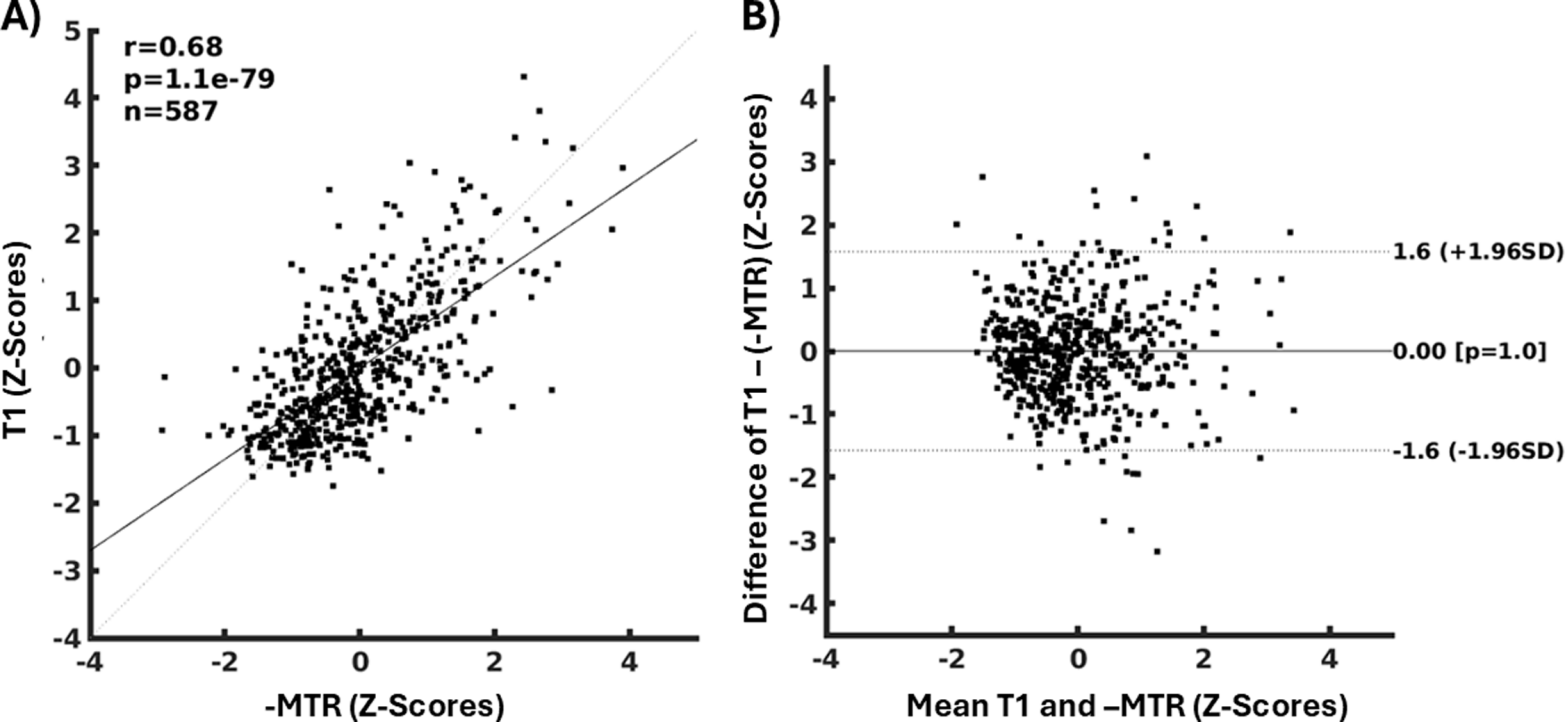
MS lesion correlation between T1 and MTR, values are standardized to Z-scores. MTR is plotted as inverse (-MTR).

The correlation plot for mean T1 and MTR values of MS lesions is shown in Fig 2A, with data standardized by z-scores due to differences in scalar units, and MTR is plotted as an inverse for better visualization. The Pearson’s correlation coefficient between lesional T1 and MTR values was r = -0.68, with p < 0.001. The Bland-Altman plot (Fig 2B) is similarly standardized by z-scores, showing the majority of data points within the level of agreements ± 1.6 z-score deviations.

### T1 and MTR correlations with MS disease metrics

Table 2 shows the correlation statistics for T1 and MTR values of lesions with EDSS, disease duration, and BPF. For T1 correlation with EDSS, no significant relationship was seen at any of the histogram stratifications. Disease duration showed a significant correlation with mode (r = 0.34, p = 0.04). BPF was significantly correlated with T1 values at 25^th^, 50^th^, and 75^th^ percentiles, and mode (r = -0.41, p = 0.01; r = -0.41, p = 0.01; r = -0.38, p = 0.02; r = -0.47, p = 0.003).

**Table 2:** Correlation of T1 and MTR values of lesions expressed as percentiles with EDSS, BPF, and disease duration.

T1	EDSS, ρ	p	duration, r	p	BPF, r	p
25th	0.25	0.127	0.31	0.06	-0.41	**0.01**
50th	0.2	0.105	0.32	0.05	-0.41	**0.01**
75th	0.29	0.079	0.30	0.07	-0.38	**0.02**
Max frequency	-0.14	0.419	-0.19	0.26	0.33	0.05
Mode	0.30	0.065	0.34	**0.04**	-0.47	**0.00**
**MTR**						
25th	-0.34	**0.03**	-0.17	0.31	0.22	0.18
50th	-0.34	**0.04**	-0.13	0.44	0.13	0.43
75th	-0.33	0.05	-0.09	0.59	0.07	0.68
Max frequency	-0.11	0.512	0.00	0.98	0.05	0.75
Mode	-0.33	**0.04**	-0.03	0.84	0.06	0.73

Abbreviations: EDSS, expanded disability status score; BPF, brain parenchymal fraction; MTR, magnetization transfer ratio.

MTR values for lesions showed significant correlation with EDSS at the 25^th^, 50^th^, and mode (r = -0.34, p = 0.03; r = -0.34, p = 0.04; r = -0.33, p = 0.04). Disease duration and BPF did not correlate with MTR at any of the quartiles, peak height, or mode.

Table 3 shows correlation of T2 lesion volumes stratified at different T1 thresholds with various MS disease metrics. Total lesion volume, without any T1 threshold, was significantly correlated with EDSS (ρ = 0.41, p = 0.01), disease duration (r = 0.49, p = 0.002), and BPF (r = -0.60, p < 0.001). Lesion volume threshold at 700 < T1 < 900 was significantly correlated with disease duration (r = 0.34, p = 0.04) and BPF (r = -0.47, p = 0.003). Lesion load threshold at 900 < T1 < 1100 was significantly correlated with EDSS (ρ = 0.42, p = 0.01), disease duration (r = 0.45, p = 0.01), and BPF (r = -0.56, p < 0.001). Lesion volume threshold at T1 > 1100 was significantly correlated with EDSS (ρ = 0.41, p = 0.01), disease duration (r = 0.45, p = 0.001), and BPF (r = -0.51, p < 0.001).

**Table 3:** Correlation of lesion volume stratified by different T1 thresholds with various measures of MS disability.

	EDSS, ρ	p	duration, r	p	BPF, r	p
Total Lesion volume (mm^3^)	0.41	**0.01**	0.49	**0.00**	-0.60	**p < 0.00**
Lesion volume (mm^3^) 700 < T1 < 900	0.23	0.16	0.34	**0.04**	-0.47	**0.00**
Lesion volume (mm^3^) 900 < T1 < 1100	0.42	**0.01**	0.45	**0.01**	-0.56	**p < 0.00**
Lesion volume (mm^3^) T1 > 1100	0.41	**0.01**	0.45	**0.00**	-0.51	**p < 0.00**

Abbreviations: EDSS, expanded disability status score; BPF, brain parenchymal fraction.

**Fig 3:**
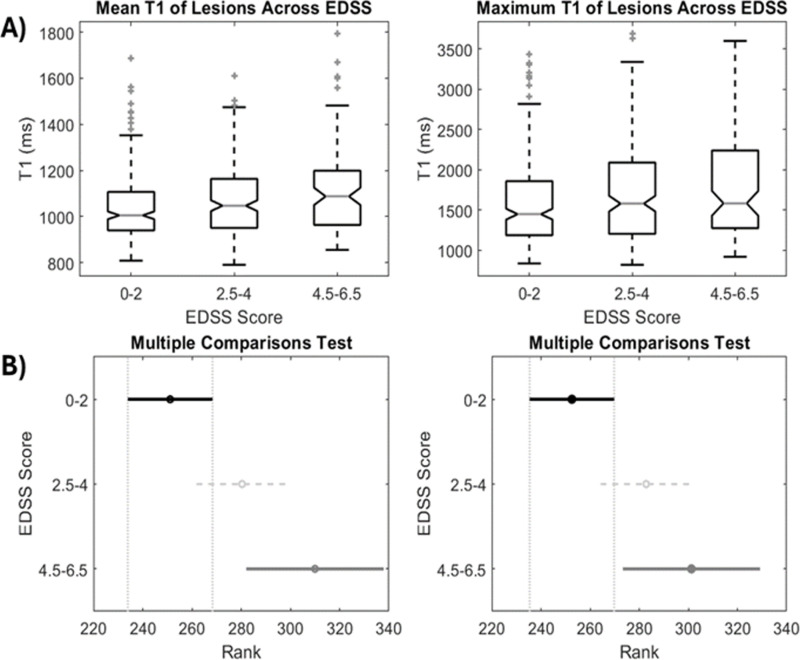
A) Comparison of mean and maximum T1 of lesions across different severity classifications of EDSS and B) Multiple Pairwise Comparisons Test of the ranks from the Kruskal-Wallis Test (±SD). The degree of overlap between lower and higher ranks of T1 values is indicated, with EDSS 0-2 and EDSS 4.5-6.5 having no overlap.

Fig 3A shows the mean T1 of lesions across different EDSS severity categories, with high T1 values seen in the worse EDSS range (p = 0.01). Fig 3B shows a plot of ranked T1 values from the Kruskal-Wallis Test against EDSS severity scores, with no overlap of standard deviations between low and high EDSS scores. The relationship between EDSS and mean T1 and MTR values in the NAWM, cortical gray matter, and thalamus are shown in Table 4. Mean T1 values in the NAWM showed a significant association with EDSS (coefficient = 0.03, pseudo R^2^ = 0.07, p = 0.03). MTR values showed significance in the thalamus (coefficient = -0.35, pseudo R^2^ = 0.06, p = 0.04).

**Table 4:** Ordinal regression analysis of T1 and MTR associations with EDSS adjusted for age.

Mean T1 (ms)	Coefficient	95% CI	χ^2^	Pseudo R^2^	p
NAWM	0.03	0.004, 0.065	10.11	0.07	**0.03**
Cortical GM	-0.01	-0.032, 0.014	5.65	0.04	0.43
Thalamus	-0.02	-0.036, 0.003	7.71	0.05	0.11
**Mean MTR (%)**					
NAWM	-0.41	-0.85, 0.05	8.03	0.05	0.08
Cortical GM	-0.35	-0.84, 0.14	6.93	0.05	0.16
Thalamus	-0.35	-0.69, -0.02	9.29	0.06	**0.04**

Abbreviations: EDSS, expanded disability status score; BPF, brain parenchymal fraction; MTR, magnetization transfer ratio; NAWM, normal appearing white matter; GM, gray matter.

## Discussion

The aim of this study was to validate a novel approach for using retroactively derived T1 values in quantifying tissue injury in MS lesions and normal-appearing tissue and correlating them with various measures of MS related disability. We also selected a commonly accepted biomarker of tissue injury, MTR, and compared the performance of retroactive T1 values with MTR. For MS lesions, the mean T1 showed a high correlation with MTR, r = 0.68, implying that both biomarkers provide complementary information regarding assessment of tissue injury. For clinical correlations, T1 values of MS lesions were stratified using different approaches for sensitivity analysis in terms of correlation with MS disease metrics. T1 values of lesions examined as percentiles did not show a significant correlation with EDSS, but it did however with BPF. MTR showed a more consistent correlation with EDSS but not with the other clinical or MRI metrics. T1 lesion volume stratified in terms of the severity of tissue injury based on T1 thresholds showed a significant correlation with all measures of disability: EDSS, disease duration, and BPF. When using ordinal regressions, T1 values in the NAWM showed a significant influence on EDSS. Taken together, we demonstrate that retroactively derived T1 maps can be used to interrogate tissue pathology at least in the MS lesions and NAWM which could be used to examine MS disease activity and progression.

Among several of the non-conventional MRI techniques, T1 relaxometry has been frequently used to interrogate tissue injury in MS. Studies have shown an increase in T1 values of MS lesions and normal appearing tissue with clinically relevant associations [9,22–25]. Using 3D IR-FLASH sequences, T1 values were significantly elevated in NAWM and cortical gray matter of MS subjects than healthy controls [23]. The peak position (mode) of T1 histograms of NAWM in MS were significantly higher than controls. The T1 histograms of the normal-appearing cortical gray matter were also shifted to the right compared to controls, i.e., higher T1 values. Histogram peak positions were also significantly higher in secondary progressive than relapsing remitting or primary progressive MS [23]. Using a high-resolution T1 mapping technique, driven equilibrium single pulse observation of T1 with high-speed incorporation of radio frequency field inhomogeneities (DESPOT1-HIFI), T1 histogram skewness in the cortical gray matter and thalamus of MS patients was higher than controls. Cortical skewness and average white matter T1 lesion values were the best predictors of cognitive performance (adjusted *R*^2^ = 0.39) [24]. Using a relatively recent technique for T1 mapping, which is now becoming a standard method for acquiring T1 maps, Magnetization Prepared 2 Rapid Acquisition Gradient Echoes (MP2RAGE), it was shown that the average T1 z-scores were higher in MS lesions than in NAWM. RRMS patients had lower average T1 z-scores in the NAWM compared to PPMS. There was a strong correlation between average T1 z-scores in MS lesions and EDSS [10]. In another study using MP2RAGE, T1W hypointense MS lesions (i.e., black holes) were stratified based on T1 values. The T1 values of visually appearing black holes were higher than T2 lesions without associated T1W hypointensity. Furthermore, lesion volumes with higher T1 values had a more robust correlation with EDSS than those with lower T1 thresholds, a finding also recapitulated when Multiple Sclerosis Functional Composite (MSFC) was used as an outcome measure [9]. T1 mapping has also been performed using high magnetic field strength (7.0 T) for characterizing fully demyelinated and partially remyelinated MS lesions [25–26]. The salient finding of the study was that the histological and 7.0 T T1 relaxometry map characteristics of remyelinating lesions were similar. The most destructive lesions as defined by the presence of paramagnetic rims (PRLs) show much longer T1 times than those without the iron rim [25]. Our data is congruent with prior studies demonstrating that T1 values are informative of tissue injury within MS lesions, and they also correlate with several of the MS disease metrics.

When comparing retroactive T1 mapping with MTR as a biomarker, we observed some differences in the strength of clinical and MRI correlations. MTR is an established biomarker of demyelination and axonal loss, as is the case with T1 relaxometry [14–15]. Previous studies have explored the relationship between MTR and T1, demonstrating that they provide complementary information when interrogating disease pathology in MS lesions and NAWM [27–30]. Using two gradient echo data sets at different repetition times to obtain T1 maps, studies have shown a high correlation between T1 values and MTR of MS lesions (r = 0.74–0.79) [15,30,31]. The correlation was equally seen in the T1W hypointense and isointense lesions [30]. Our data are similar to previous studies demonstrating a high correlation between T1 times and MTR within MS lesions (r = 0.68), corroborating our retroactive methodology. Hence, both MTR and retroactive T1 mapping could be used as complementary techniques, the advantage of the latter approach is that no additional scanning is required. In terms of correlations between EDSS scores and lesional MTR, prior studies show a modest correlation, r = -0.27 to -0.32 [32–34]. In our dataset, we also observed similar results, r = -0.33 with lesional MTR and mode. We did not observe any significant correlations between MTR values of lesions and disease duration or BPF. When T1 values of lesions were expressed in terms of percentiles, we did not observe any significant correlations between T1 and EDSS, but they were seen in the MTR data. However, significant correlations were seen between BPF and T1 of lesions, but not MTR. Beyond MS lesions, prior studies show an association between EDSS scores and MTR in the NAWM, thalamus, and NAGM [35–37]. This was only observed for the thalamus in our dataset. A plausible explanation for these discrepancies in the correlation statistics between T1 and MTR could be cohort differences, therapeutic interventions, or sample size. All subjects in our study were on a high-efficacy therapy for MS and were stable clinically and by MRI criteria prior to enrollment, which was not the case in prior studies. Subjects with a more active or progressive disease course could affect correlation statistics in the model. Another explanation could be that although T1 and MTR have some collinearity in interrogating tissue injury, they are not however entirely congruous techniques. Both techniques gauge different aspects of tissue pathology. T1 values are influenced by the degree of free water and iron content of the tissue whereas MTR values are determined by water protons bound to macromolecules and their efficiency of transfer to the free pool [38–39]. Different non-conventional MR techniques may be differentially correlated with different types of clinical or MRI biomarkers of disability depending on their sensitivity for various aspects of tissue pathology.

Based on our results, retroactive T1 mapping may be best suited for monitoring the status of tissue injury in MS lesions and NAWM. Disease worsening in MS is associated with increasing lesion number, size, and alterations in the NAWM [34]. Recent studies have provided evidence for ongoing chronic inflammatory activity in MS lesions. These chronic active lesions (CALs) are described as slowly evolving/expanding lesions (SEL) over time on conventional MRI scans, and on susceptibility-weighted imaging, as paramagnetic rim lesions (PRLs) [40–41]. Both types of lesions (SELs and PRLs) are correlated with clinical and MRI disease progression [40–42]. Retroactive T1 mapping may be an effective and practical method for monitoring SELs as they are readily seen on T1 maps. Prior studies have shown a reduction in T1W hypointensity and MTR in the more destructive MS lesions (persistent black holes), and a higher proportion of SELs and lower baseline MTR values of SELs were independent predictors of EDSS score worsening at 9-year follow-up [40,43]. Similarly, SELs were examined in the pooled population of two phase III, multicenter, randomized, double-blinded clinical trials, OPERA I and OPERA II [44]. SELs showed a significant decrease in the normalized T1-weighted hypointensity from baseline to weeks 96 and 120 in relapsing and progressive MS patients. Compared to non-SELs, there was a significant decrease in T1-weighted hypointensity at all longitudinal timepoints from baseline to weeks 96 and 120 in these cohorts [44]. Instead of monitoring lesion T1W hypointensities, T1 values would be a more accurate method for examining SELs because they provide a better estimation of tissue injury, and the ambit of T1 values encompasses a wider range to detect more subtle changes in tissue injury over time.

There are a few limitations to this study. The sample size is small, with n = 38. However, MS subjects in aggregate contributed to 587 lesions for analysis, which may be sufficient and apropos to the main goal of the study, which was to interrogate MS lesion pathology and its relationship to MS disability metrics. The sample size is also similar to previously published literature examining non-conventional imaging techniques to interrogate MS lesion pathology [9,29,30]. We only examined subjects that were clinically and radiologically stable to exclude the effects of steroids or recent changes in therapies on T1 relaxation, which is sensitive to alteration in the water content, especially of lesions. Also, for validation purposes of a novel methodology, stable MS lesions would be an appropriate initial phase to start. However, our preliminary work shows that retroactive T1 values of active lesions are significantly higher than chronic lesions, hence our methodology can capture acute lesions as well, a topic for future investigations. We only used MPRAGE type of images for conversion to T1 maps. However, our pipeline can easily be adapted to any type of high-resolution anatomical T1W images, including 3D-spoiled gradient echo (3D-SPGR), 3D fast low-angle shot (3D-FLASH), and T1 spine echo (SE) (preliminary work).

## Conclusions

Our findings demonstrate that retroactive T1 mapping is a reliable method for stratifying tissue injury in MS, and it is correlated with clinical and MRI measures of MS related disability. The main advantage of retroactive T1 mapping is that it uses high resolution MPRAGE images that have already been acquired. To further test the usefulness of retroactive T1 mapping, future studies using a larger and longitudinal dataset and other types of high-resolution T1W sequences across different scanners would further provide corroborating evidence. A more diverse patient cohort, in terms of demographics, disease activity, and course, would further test the applicability of retroactive T1 mapping as a robust biomarker of MS disease state.

## Supporting information

S1 Data Data supporting information. (XLSX)
